# FM-FCN: A Neural Network with Filtering Modules for Accurate Vital Signs Extraction

**DOI:** 10.34133/research.0361

**Published:** 2024-05-10

**Authors:** Fangfang Zhu, Qichao Niu, Xiang Li, Qi Zhao, Honghong Su, Jianwei Shuai

**Affiliations:** ^1^ Department of Physics, and Fujian Provincial Key Laboratory for Soft Functional Materials Research, Xiamen University, Xiamen 361005, China.; ^2^ National Institute for Data Science in Health and Medicine, and State Key Laboratory of Cellular Stress Biology, Innovation Center for Cell Signaling Network, Xiamen University, Xiamen 361005, China.; ^3^ Vitalsilicon Technology Co. Ltd., Jiaxing, Zhejiang 314006, China.; ^4^ School of Computer Science and Software Engineering, University of Science and Technology Liaoning, Anshan 114051, China.; ^5^ Yangtze Delta Region Institute of Tsinghua University, Zhejiang, Jiaxing 314006, China.; ^6^ Wenzhou Institute, University of Chinese Academy of Sciences, Wenzhou 325001, China.; ^7^ Oujiang Laboratory (Zhejiang Lab for Regenerative Medicine, Vision and Brain Health), Wenzhou 325001, China.

## Abstract

Neural networks excel at capturing local spatial patterns through convolutional modules, but they may struggle to identify and effectively utilize the morphological and amplitude periodic nature of physiological signals. In this work, we propose a novel network named filtering module fully convolutional network (FM-FCN), which fuses traditional filtering techniques with neural networks to amplify physiological signals and suppress noise. First, instead of using a fully connected layer, we use an FCN to preserve the time-dimensional correlation information of physiological signals, enabling multiple cycles of signals in the network and providing a basis for signal processing. Second, we introduce the FM as a network module that adapts to eliminate unwanted interference, leveraging the structure of the filter. This approach builds a bridge between deep learning and signal processing methodologies. Finally, we evaluate the performance of FM-FCN using remote photoplethysmography. Experimental results demonstrate that FM-FCN outperforms the second-ranked method in terms of both blood volume pulse (BVP) signal and heart rate (HR) accuracy. It substantially improves the quality of BVP waveform reconstruction, with a decrease of 20.23% in mean absolute error (*MAE*) and an increase of 79.95% in signal-to-noise ratio (*SNR*). Regarding HR estimation accuracy, FM-FCN achieves a decrease of 35.85% in *MAE*, 29.65% in error standard deviation, and 32.88% decrease in 95% limits of agreement width, meeting clinical standards for HR accuracy requirements. The results highlight its potential in improving the accuracy and reliability of vital sign measurement through high-quality BVP signal extraction. The codes and datasets are available online at https://github.com/zhaoqi106/FM-FCN.

## Introduction

Noncontact techniques for monitoring and diagnosing vital signs have gained significant attention in the field of medical diagnostics field. These techniques, including microwave doppler [1], ballistocardiogram [2], and remote photoplethysmography (rPPG) [3,4], allow for the measurement and monitoring of vital signs such as heart rate (HR), respiratory rate, and blood oxygen saturation without direct contact with the patient’s skin. The use of these techniques offers several advantages, such as convenience and comfort for patients, as well as a reduced risk of infection transmission. Among these techniques, rPPG is particularly noteworthy because of its ability to utilize ubiquitous devices like smartphones or computer cameras without the need for specialized sensors. By analyzing changes in skin color, rPPG can accurately capture HR [3], blood flow [5], and other vital sign information [6], while also being capable of recognizing an individual’s emotional state [7]. The development of rPPG technology brings forth exciting possibilities in healthcare, mental health, and various innovative applications [8,9].

Accurate vital sign extraction and analysis rely on high-quality blood volume pulse (BVP) waveforms. For example, HR variability is determined by the variations in HR cycles, while features extracted from the BVP waveform are used to assess blood pressure. However, existing methods encounter challenges when working with face videos that exhibit facial deformations, illumination variations, and camera sensor noise [10]. These factors can result in inaccurate estimation of the BVP signal. Therefore, there is an urgent need to develop innovative rPPG techniques capable of accurately extracting BVP waveforms even in the presence of noise. The development of such techniques will enable the computation of a wider range of physiological features.

There are 2 main approaches to implementing rPPG: classical-theory-based techniques and deep-learning-based techniques. Classical-theory-based techniques utilize signal processing methods such as blind source separation (BSS) [11], plane-orthogonal-to-skin (POS) algorithm [12], independent component analysis (ICA) [13], constrained ICA [14], and local group invariance (LGI) [15] to extract the BVP signal from the captured video frames. These techniques often make assumptions about noise characteristics and simplifications to effectively reduce noise and achieve accurate vital sign measurements under ideal conditions. Commonly used classical techniques include filters, differentials, and statistical methods, which aim to remove irrelevant signal and noise artifacts while preserving relevant physiological information. In some cases, machine learning techniques are incorporated to improve the accuracy of extracted vital signs [11]. However, classical techniques may not yield satisfactory results when dealing with complex noise sources or nonlinear variations in real-world scenarios. Factors such as environmental illumination variations, facial expressions, and motion can introduce significant noise contamination into the rPPG signal, making it challenging to achieve accurate and reliable vital sign monitoring. To overcome these limitations, deep-learning-based rPPG techniques have emerged as a promising alternative. These techniques leverage deep neural networks such as convolutional neural networks (CNNs) [16,17], generative adversarial network [18,19], and long short-term memory (LSTM) [7] to learn complex features directly from raw video frames and accurately capture the signal area to enhance weak feature signals. Deep-learning-based approaches have demonstrated improved performance in handling motion environments and nonlinear variations, making them more robust for BVP extraction in certain real-world scenarios.

Although deep learning networks have exhibited impressive performance in spatial signal decomposition and reconstruction, they often face challenges in effectively utilizing information in the time domain, particularly in the context of physiological signal processing. In this work, we introduce a new network, filtering module fully convolutional network (FM-FCN), designed to augment the model’s ability to diminish unwanted signals. This enhancement not only optimizes performance in rPPG tasks but also shows promising potential in spatiotemporal data analysis [20,21] and prediction [22]. The primary contributions of this work can be summarized as follows.

1. Introducing FM tailored specifically for processing physiological signals. FM serves as an innovative bridge between deep learning and signal processing techniques, effectively eliminating unwanted interference and thereby enhancing the signal-to-noise ratio (SNR) of the signals.
2. Incorporating FCN that is particularly suited for periodic physiological signals. FCN directly outputs multiperiodic of BVP waveforms, thereby enhancing the efficiency of FM-FCN and enabling the exploitation of temporal correlation. By using FCN instead of fully connected layers, we considerably reduce the number of parameters and facilitate weight sharing in the temporal dimension, leading to enhanced signal reconstruction accuracy.
3. Comprehensive establishment of evaluation metrics for rPPG technology, encompassing indicators for both BVP waveform quality and HR accuracy. The assessment of BVP waveform quality is conducted on the basis of the time-domain *SNR*, which mitigates the risk of erroneously attributing noise energy as a signal capability in frequency-domain methods. HR accuracy is evaluated according to the ANSI/AAMI EC13:2002 standard [4], which holds more relevance in medical applications.
  The subsequent sections of this paper are organized as follows. The review of the related work is presented in Related Work with details. Methodology outlines the proposed method. Experiment Results presents the experimental results. A detailed discussion is in Discussion. Finally, Conclusion and Future Works concludes the study and offers future directions for researchers and practitioners.

## Comprehensive framework for rPPG

**Fig. 1.**
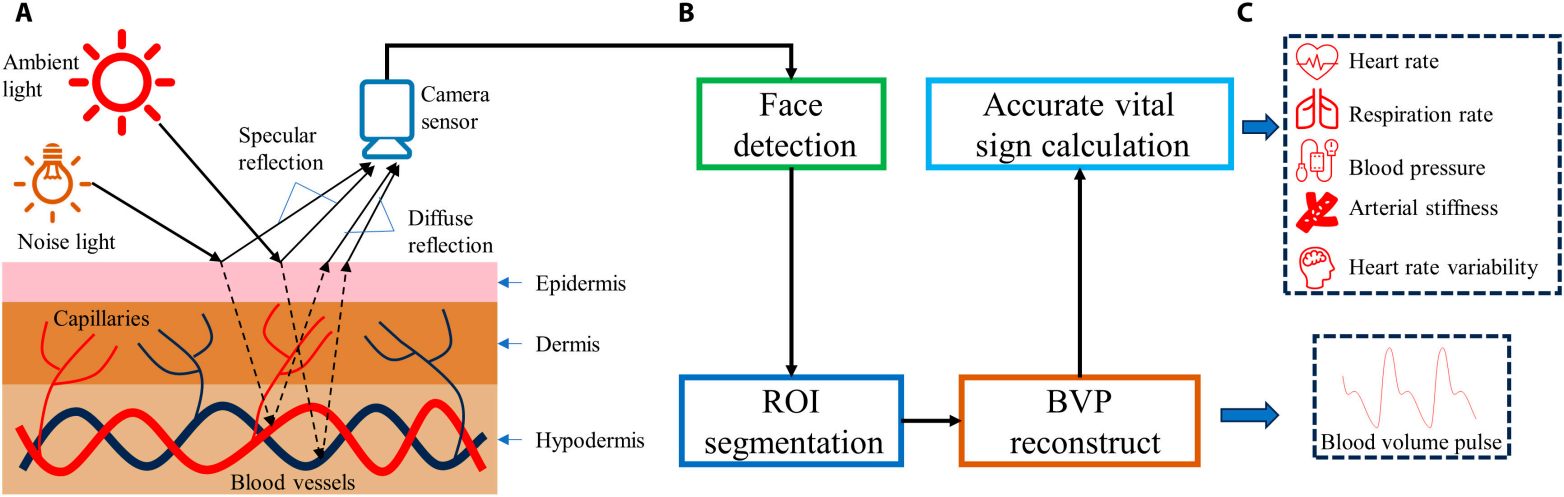
A comprehensive framework for rPPG techniques. (A) Illustration of the photoelectric signal acquisition related to BVP. (B) Overview of the general process involved in rPPG signal processing. (C) Vital signs information available based on rPPG.

The absorption of light by blood vessels exhibits periodic changes due to the rhythmic contractions of the heart. As illustrated in Fig. 1, this subtle fluctuation is captured by a camera, resulting in BVP signal. However, this BVP signal is inherently weak and susceptible to various interferences, including fluctuations in ambient light, flashing lights, and changes in facial orientation. Therefore, it is essential to denoise and enhance the SNR of the BVP signal to accurately extract vital signs from videos. Typically, the processing of rPPG technology involves 4 main steps: face detection, ROI segmentation, BVP signal reconstruction, and vital sign calculation. The signal acquired by the camera sensors can be mathematically represented as follows.

$$C\left(t\right)=\left[\begin{array}{cccc} I_{1,1}\left(t\right) & I_{1,2}\left(t\right) & \cdots & I_{1,N}\left(t\right)\\ I_{2,1}\left(t\right) & I_{2,2}\left(t\right) & \cdots & I_{2,N}\left(t\right)\\ \vdots & \vdots & \ddots & \vdots \\ I_{M,1}\left(t\right) & I_{M,2}\left(t\right) & \cdots & I_{M,N}\left(t\right) \end{array}\right]$$ (1)

where *C*(*t*) denotes a matrix of the intensity value on a color channel of *M* × *N* image during time *t*. *I*_*i*,*j*_(*t*) refers to the intensity value of a specific pixel located at row *i* and column *j* during time *t*. To account for the presence of noise or irrelevant signals, we decompose each pixel using Eq. 2.

$$I_{i,j}\left(t\right)=s_{i,j}\left(t\right)+n_{i,j}\left(t\right)$$ (2)

where *s*_*i*,*j*_(*t*) and *n*_*i*,*j*_(*t*) represents the intensity values of signal and noise at row *i* and column *j* during time *t*, respectively.

## Filtering module

Filters play a crucial role in signal processing, as they selectively extract specific frequency components or features from raw signals while eliminating unwanted portions. Two commonly used types of filters are frequency domain filters and adaptive filters. Frequency domain filters are designed to suppress undesired frequency signals, while adaptive filters excel at filtering out random interference or noise with specific statistical properties. However, both types of filters rely on convolution calculations, where the input signal is convolved with the filter coefficient. A general formula for a filter can be expressed as follows.

$$f\left(x;h\right)=\sum_{k=0}^{K}h\left(k\right)\times x\left(t-k\right)$$ (3)

where *f*(*x*; *h*) represents the filtered output signal using filter coefficient *h*, and *x*(*t*) denotes the input signal, while *h*(*k*) represents the impulse response of the filter. The summation is performed over a range of *k* values. The impulse response provides information about the behavior of the filter and determines how the filter responds to different signals, particularly those with varying frequencies.

**Fig. 2.**
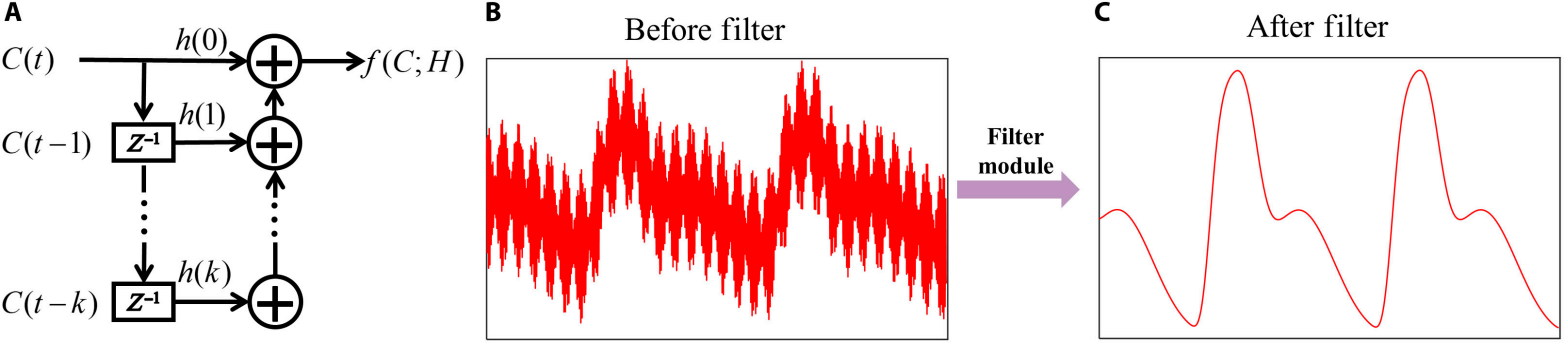
Introduction to FM. (A) General structure of FM. (B) Signals before undergoing the FM processing. (C) Signals after undergoing the FM processing.

Physiological signals often exhibit inherent periodicity and statistical regularity, making filtering techniques a widely utilized tool in signal processing. However, deep-learning-based approaches often overlook these valuable characteristics. Here, we propose an FM specifically designed for integration into convolutional networks, as depicted in Fig. 2A. This module represents a generalized structure of FM, where the value of each pixel is influenced by its historical counterparts. By incorporating FM as a filtering mechanism, unwanted noise and signals can be effectively removed, thereby facilitating the extraction of the desired physiological signals from the original input, as visually demonstrated in Fig. 2C derived from Fig. 2B. The filtering process is mathematically expressed in Eq. 4, and Eq. 5 provides a detailed explanation of the pixel-wise filtering operation.

$$f\left(C;\;H\right)\;=\;\left[\begin{array}{cccc} f\left(I_{1,1};\;h_{1,1}\right) & f\left(I_{1,2};\;h_{1,2}\right) & \cdots & f\left(I_{1,N};\;h_{1,N}\right)\\ f\left(I_{2,1};\;h_{2,1}\right) & f\left(I_{2,2};\;h_{2,2}\right) & \cdots & f\left(I_{2,N};\;h_{2,N}\right)\\ \vdots & \vdots & \ddots & \vdots \\ f\left(I_{M,1};\;h_{M,1}\right) & f\left(I_{M,2};\;h_{M,2}\right) & \cdots & f\left(I_{M,N};\;h_{M,N}\right) \end{array}\right]$$ (4)

$$f\left(I_{i,j};\;h_{i,j}\right)=\sum_{k=0}^{K}h_{i,j}\left(k\right)\times I_{i,j}\left(t-k\right)$$ (5)

where *h_i,j_*(*k*) represents the impulse response of the filter applied to the pixel located at position (*i*, *j*) during time *t*.

FM can be implemented with various filter structures, such as finite impulse response, infinite impulse response, median filter, or other adaptive filters. The flexibility of these modules allows them to be embedded in any node of a deep learning network, enabling each node to select a specific filter structure based on its specific requirements. In this work, we demonstrate the use of finite impulse response structures to design FMs.

## FM-FCN

**Fig. 3.**
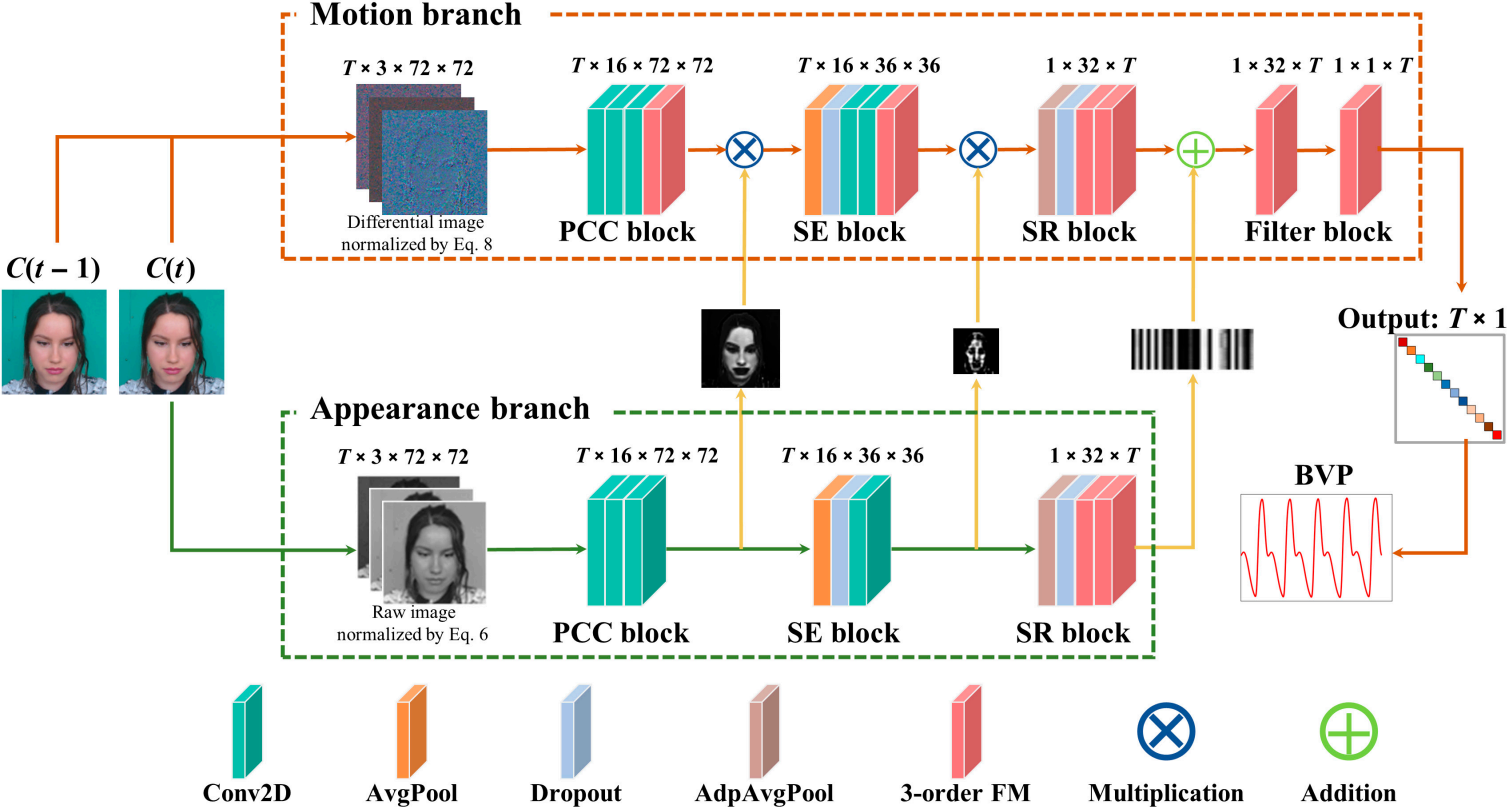
Architecture of FM-FCN. PCC block clusters principal component through extraction from large-scale images. SE block extracts signals from small-scale images. SR block flattens and extracts signals at an SR level. Filter block denoises the signal.

The overall architecture of FM-FCN is depicted in Fig. 3. It consists of 2 primary branches: the motion branch and the appearance branch. The motion branch is responsible for extracting the physiological signal, while the appearance branch is utilized to estimate spatial weight information.

The appearance branch of FM-FCN is composed of 3 blocks: the principal component clustering (PCC) block, the signal extraction (SE) block, and the superresolution (SR) block. PCC block utilizes 2 2D CNN layers to cluster the principal components of large-scale images. This helps in capturing significant patterns and generating a mask. Similarly, SE block extracts signals from small-scale images using an average pooling layer, a dropout layer, and 2 2D CNN layers. It also generates a mask. SR module further processes the generated 1D SR signal. It uses various layers such as average pooling, adaptive pooling, dropout, and 3-order FMs. The aim of SR block is to generate a mask that facilitates the extraction of relevant weights for enhancing the objective signal. The primary aim of the appearance branch is to encapsulate spatial attributes associated with BVP variations. Considering that motion between adjacent frames is generally minimal, this branch enhances the delineation of edges between the face and the background through the application of averaging techniques. This method facilitates the accurate extraction of facial contours while concurrently diminishing the impact of random noise. The refined input for the appearance branch is mathematically depicted in Eq. 6.

$$A\left(t\right)=\left(\text{Ave}\left(t\right)-\mu_{t}\left(\text{Ave}\left(t\right)\right)\right)/\sigma_{t}\left(\text{Ave}\left(t\right)\right)$$ (6)

$$\text{Ave}\left(t\right)=\left(C\left(t\right)+C\left(t-1\right)\right)/2$$ (7)

where *A*(*t*) represents the input appearance, Ave(*t*) is a frame generated by averaging 2 adjacent frames *C*(*t*) and *C*(*t* − 1), *μ*(Ave(*t*)) denotes the average value of a frame, and *σ*(Ave(*t*)) is the standard deviation (*SD*) of a frame.

The motion branch in our system exhibits a structure resembling that of the appearance branch, with the addition of a filter block and a 3-order FM at the end of PCC and SR block. The primary objective of the motion branch revolves around extracting BVP waveform. To accomplish this, it is imperative to integrate an FM module or block within each block, prior to attaining the final BVP waveform output. This particular FM plays a critical role in mitigating noise and ensuring accurate extraction of BVP waveform. Multiple FM modules have been observed to simulate the characteristics of band-pass or band-stop filters. Furthermore, drawing on insights from [35], differential operations are applied to the input of the motion branch to effectively eliminate common-mode interference and irrelevant dc signals. This process is streamlined into Eqs. 8 and 9, which calculate the difference between adjacent frames along the temporal axis of the raw video frames, thereby eliminating low-frequency interference.

$$M\left(t\right)=\left(D\left(t\right)-\mu \left(D\left(t\right)\right)\right)/\sigma_{t}\left(D\left(t\right)\right)$$ (8)

$$D\left(t\right)=C\left(t\right)-C\left(t-1\right)$$ (9)

where *M*(*t*) represents the input motion, *D*(*t*) denotes the frame difference obtained by subtracting the previous frame *C*(*t* − 1) from the current frame *C*(*t*).

Finally, we replace the fully connected layers with FCN to obtain 2 notable advantages. First, physiological signals commonly exhibit periodic patterns, and by utilizing filters with longer periods during signal processing, we can effectively leverage the temporal correlation of the signal for a more precise feature reconstruction. Second, the inclusion of FCN enables parameter reduction and weight sharing along the temporal dimension. In summary, FM-FCN is specifically designed for the extraction of physiological signals, achieving an optimal fusion of temporal and spatial information.

## Spatiotemporal feature fusion

**Fig. 4.**
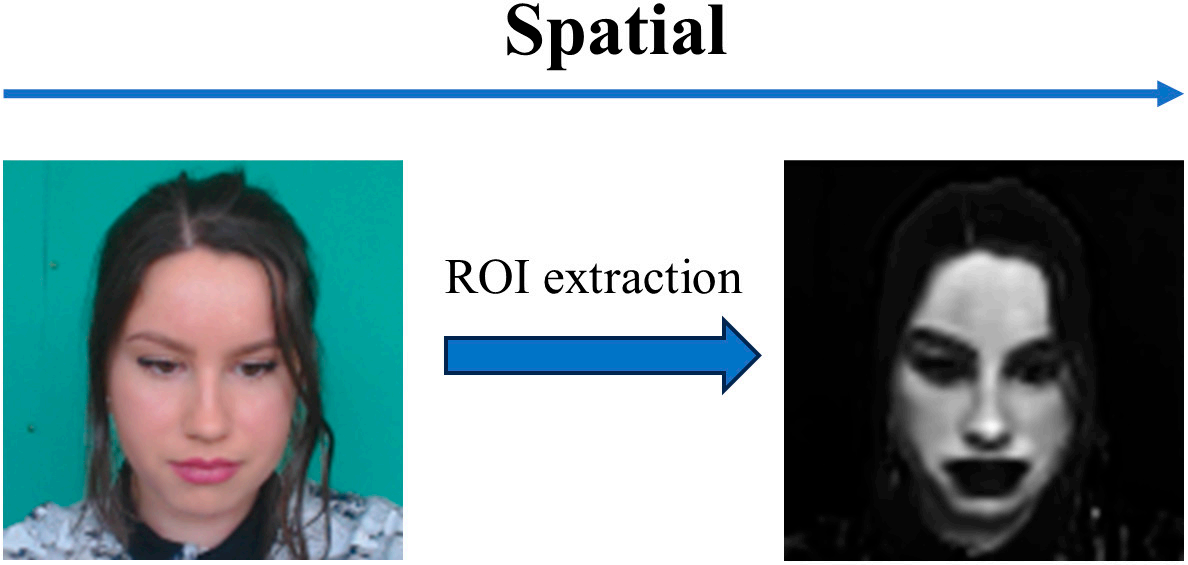
Example of spatial ROI extraction using deep learning networks.

Deep learning networks have shown remarkable effectiveness in accurately delineating facial contours, as illustrated in Fig. 4. This capability is crucial for the precise identification of the ROI, predominantly areas of the facial skin, which are vital for subsequent analyses. By leveraging these identified ROIs, it is possible to analyze periodic variations in the intensity of reflected light at specific wavelengths from video footage, facilitating the extraction of the BVP signal. Therefore, deep learning techniques are the ideal choice for precisely identifying relevant ROIs and serve as a robust foundation for extracting BVP signals.

**Fig. 5.**
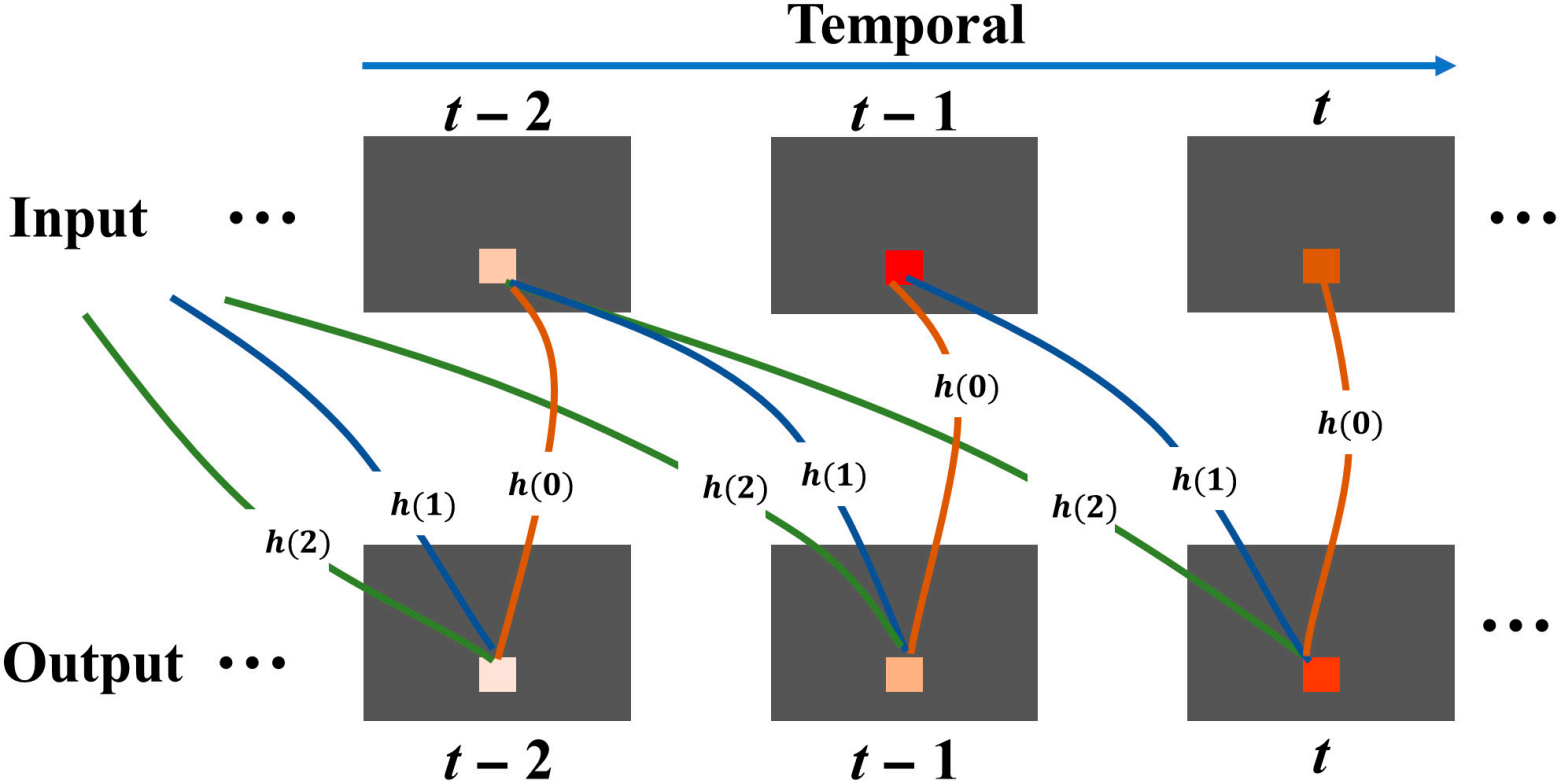
Denoising of multiframe images through the utilization of spatiotemporal features. The filtering method FM is applied to the input images, resulting in output images with diminished noise in the pixel values.

However, the amplitude of the intensity change corresponding to the BVP signal is very weak compared to the intensity change caused by other factors in the videos. In other words, the weak BVP signal would be overwhelmed and interfered with by various noises. To address these challenges, we propose an FM to eliminate noise and enhance BVP signal. Considering that physiological signals often exhibit certain characteristics such as periodicity and slow variability, FM is used to fuse classical filtering techniques with deep learning networks, as illustrated in Fig. 5. It applies temporal filtering to the current frame image and multiple previous frames, effectively incorporating temporal information. In addition, FM can be envisaged as a 1D convolution kernel operating along the temporal axis, with its parameters initiated and defined following a similar approach as with other kernels. Consequently, through the training process, a set of filtering parameters is derived, leading to an enhanced *SNR* of the output BVP waveform. This fusion of classical filtering techniques with deep learning networks via FM provides a promising approach for enhancing the extraction of BVP signals, mitigating the effects of noise, and improving the overall quality of signal reconstruction.

## Results

### Datasets and training settings

We incorporate 4 distinct datasets for the experiment, namely, pulse rate estimation database (PURE) [23], University of Bourgogne Franche-Comté rPPG (UBFC-rPPG) [24], remote learning affect and physiologic dataset (RLAP) [25], COHFACE [26]. Each of these datasets provides unique information and features that are critical for the overall experiment. For a comprehensive overview of these databases, including their respective characteristics and properties, please refer to Table 1.

**Table 1.** A detailed overview of the datasets

Dataset size	Dataset name	Individual number	Frame rate	Duration	Resolution
Small scale	PURE [23]	59	30 fps	≈1 min	640 × 480
	UBFC-rPPG [24]	42	30 fps	≈2 min	640 × 480
Large scale	RLAP [25]	728	30 fps	1–7 min	1,920 × 1,080
	COHFACE [26]	160	20 fps	≈1 min	640 × 480

The extraction of BVP waveform is conducted using a unit of 256 frames, equivalent to approximately 8 s of videos. The primary experiments can be divided into 3 parts: qualitative comparison of BVP waveform restoration quality, comparison of generalization ability based on small-scale datasets, and comparison of robustness based on large-scale datasets. In the first 2 experiments, training is performed on UBFC-rPPG dataset, while testing is carried out on PURE dataset. The third experiment entails training on a subset of RLAP dataset and testing on the remaining RLAP dataset, as well as all other datasets, to evaluate the robustness of various methods.

In addition, in the subsequent experiments, unless specifically stated otherwise, “referenced BVP” (rBVP) refers to the BVP waveforms recorded by medical devices present in the dataset, typically derived from finger clip pulse oximeters, representing the gold standard and data labels. “Referenced HR (rHR)” denotes the HR values calculated on the basis of the rBVP. Similarly, “estimated BVP” (eBVP) refers to the BVP waveforms extracted from video data using rPPG methods, and “estimated HR (eHR)” represents the HR values calculated on the basis of the eBVP.

### Evaluation metrics

To evaluate the effectiveness of FM-FCN in extracting BVP waveform, 3 essential metrics are utilized in this work. The first metric, mean absolute error (*MAE*) as shown in Eq. 10, provides a direct assessment of waveform quality by measuring the average magnitude of differences between the eBVP waveforms and the rBVP waveforms. The second metric, Pearson correlation coefficient (*R*) represented by Eq. 11, evaluates the linear relationship between the eBVP waveforms and the rBVP waveforms. It offers insights into the similarity in shape and trend, providing an indication of enhancement fidelity. To quantify *SNR*, we use a time-domain approach described by Eq. 12 instead of the commonly used approach mentioned in [27]. This alternative approach is more appropriate as it takes into consideration that the first harmonic of the calculated signal’s spectra may represent noise energy rather than the actual BVP signal energy. This metric enables an accurate evaluation of the clarity of BVP signal despite potential noise interference. The definitions of these 3 metrics are provided below.

$$MAE=\frac{1}{N}\sum_{i=1}^{N}\left|{\hat{y}}_{i}-y_{i}\right|$$ (10)

$$\begin{array}{c} R=\\ \frac{N\times \sum_{i=1}^{N}\left(y_{i}\times {\hat{y}}_{i}\right)-\sum_{i=1}^{N}y_{i}\times \sum_{i=1}^{N}{\hat{y}}_{i}}{\sqrt{N\times \sum_{i=1}^{N}y_{i}^{2}-{\left(\sum_{i=1}^{N}y_{i}\right)}^{2}}\times \sqrt{N\times \sum_{i=1}^{N}{\hat{y}}_{i}^{2}-{\left(\sum_{i=1}^{N}{\hat{y}}_{i}\right)}^{2}}} \end{array}$$ (11)

$$SNR=10\times {\text{log}}_{10}\left(\sum_{i=1}^{N}y_{i}^{2}/\sum_{i=1}^{N}{\left(y_{i}-{\hat{y}}_{i}\right)}^{2}\right)$$ (12)

where *N* represents the total number of samples, $\hat{y}$ represents the calculated value, and *y* represents the reference value.

In addition to *MAE* and *R*, several other metrics are utilized to evaluate the accuracy of the eHR compared to the rHR. These metrics include the mean relative error (*MRE*), the accuracy rate (*ACC*), and the error *SD*. *MRE* calculates the average percentage difference between the estimated and reference HRs, providing a measure of the relative error in HR estimation. *SD *quantifies the standard deviation of errors between the estimated and reference HRs, giving an indication of the robustness in the HR estimations. *ACC* measures the percentage of eHRs that meet the ANSI/AAMI EC13-2002 standard [28]. This standard defines a tolerance of ±10% or ±5 beats/min (bpm) (whichever is greater) for the HR error. *ACC* provides an evaluation of the algorithm’s ability to accurately estimate HR values within the specified limits. The definitions of these 3 metrics are presented as follows.

$$MRE=\frac{1}{N}\times \sum_{i=1}^{N}\left|\left({\text{eHR}}_{i}-{\text{rHR}}_{i}\right)/{\text{rHR}}_{i}\right|\times 100\%$$ (13)

$$SD=\sqrt{\frac{\;\;\sum_{i=1}^{N}\left({\text{err}}_{i}-\mu \left(\text{err}\right)\right)}{N}}$$ (14)

$$ACC=\frac{ATE}{ATE+UTE}$$ (15)

where *the* variable *err* corresponds to the absolute error between the HR values (|eHR − rHR|) and *μ*(err) signifies the mean of the *err*. *ATE* indicates the count of HR errors within the allowable total error range, which adheres to the standard defined in [28]. Conversely, *UTE* represents the count of HR errors exceeding the allowable total error limit, failing to meet the standard defined in [28].

### Qualitative analysis of BVP waveform restoration

**Fig. 6.**
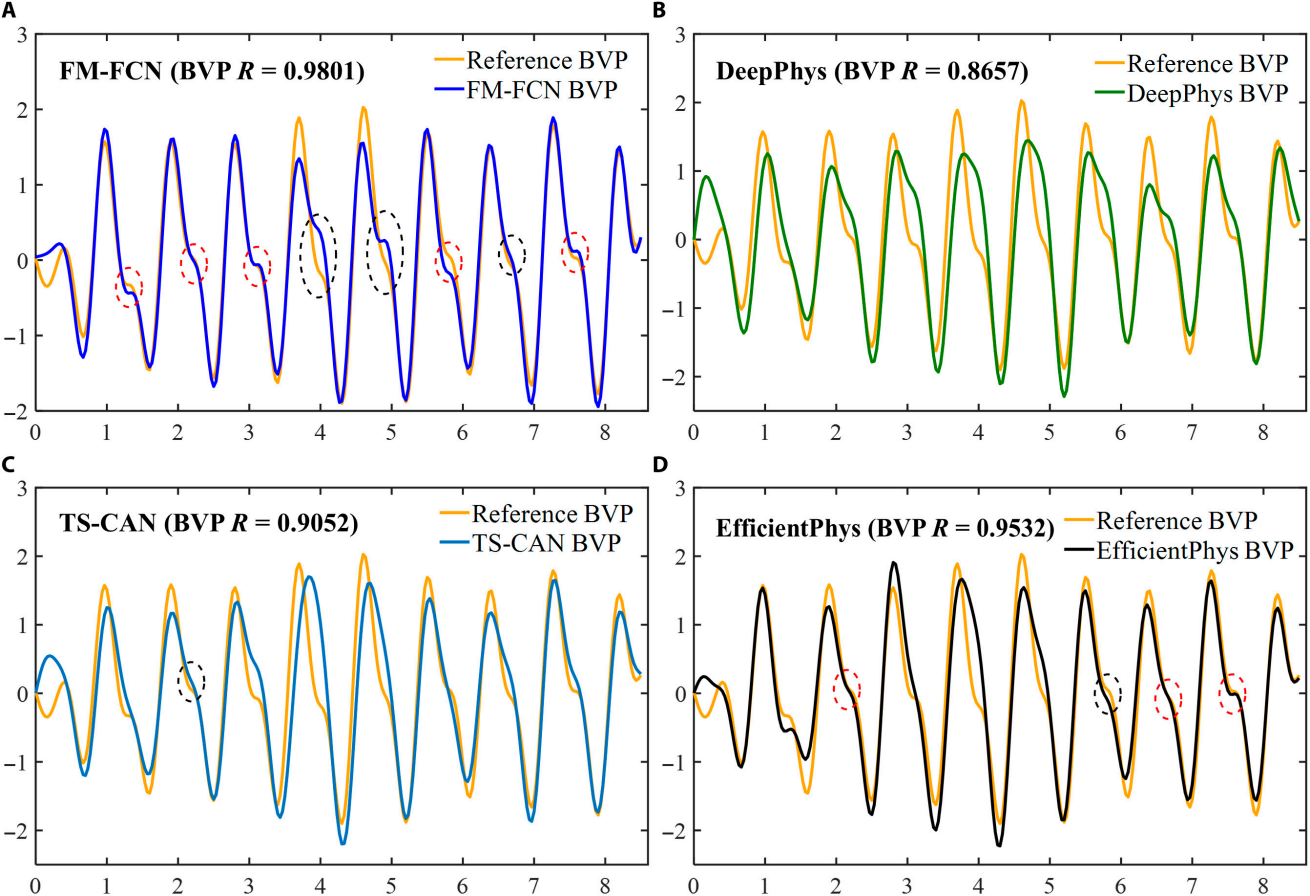
Qualitative analysis of BVP waveforms on PURE datasets. BVP waveforms obtained through various methods: (A) FM-FCN (*R* = 0.9801), (B) DeepPhys (*R* = 0.8657), (C) TS-CAN (*R* = 0.9052), and (D) EfficientPhys (*R* = 0.9532). The oval dashed line box emphasizes the well-preserved detailed features, where red indicates optimal restoration and black indicates rudimentary restoration.

BVP waveform is essential for obtaining various vital signs information, including HR, blood pressure, blood oxygen levels, and HR variability. Therefore, it is important for an algorithm to effectively reconstruct the BVP waveform to extract other vital signs data reliably. Figure 6 clearly demonstrates that BVP waveform extracted by FM-FCN has the highest correlation coefficient of 0.98, indicating a strong relationship between the reconstructed waveform and the reference BVP waveform. Moreover, BVP waveform reconstructed by FM-FCN successfully captures the second characteristic wave, which is critical for blood pressure calculation. In contrast, DeepPhys and TS-CAN fail to extract this characteristic wave, while EfficientPhys only partially captures it. This emphasizes the superior performance of FM-FCN in preserving the temporal and spatial characteristics of the secondary peak within BVP waveform.

### Evaluation on small datasets

**Fig. 7.**
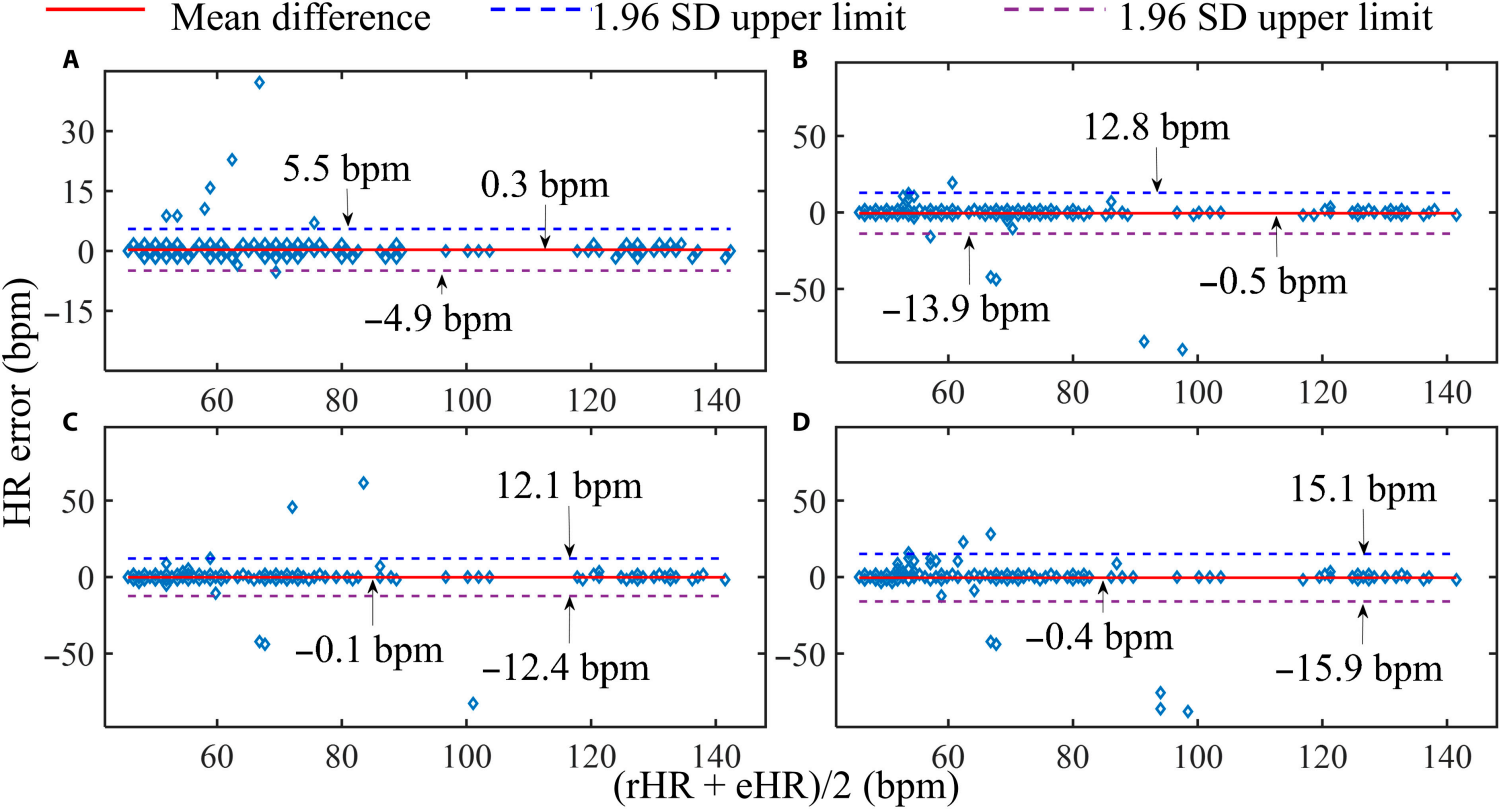
Bland–Altman consistency analysis on small datasets. The subplots (A) to (D) correspond to the evaluations conducted on FM-FCN, DeepPhys, TS-CAN, and EfficientPhys, respectively.

In this experiment, we aim to evaluate the generalization capability of different methods in the field of rPPG technology. The evaluation is conducted by training the algorithms on UBFC-rPPG dataset and assessing their performance on PURE dataset. Figure 7 shows the Bland–Altman plot analysis to quantify the agreement between the estimated and the reference HR values. Here, the difference between the HR values (*y* axis) is plotted against the mean of the HR values (*x* axis) with 95% limits of agreement (*LoA* = mean ± 1.96 × SD) that are presented with dashed lines. FM-FCN achieves HR difference within the range of [−4.92, 5.51] bpm. In contrast, DeepPhys, TS-CAN, and EfficientPhys exhibit lower accuracy, with HR errors falling within the ranges of [−13.87, 12.83], [−12.41, 12.11], and [−15.94, 15.12] bpm, respectively. The width of the *LoA* interval of FM-FCN is reduced by 57.56% to 66.52% compared with alternative methods. These findings indicate that FM-FCN outperform DeepPhys, TS-CAN, and EfficientPhys in terms of HR accuracy.

**Fig. 8.**
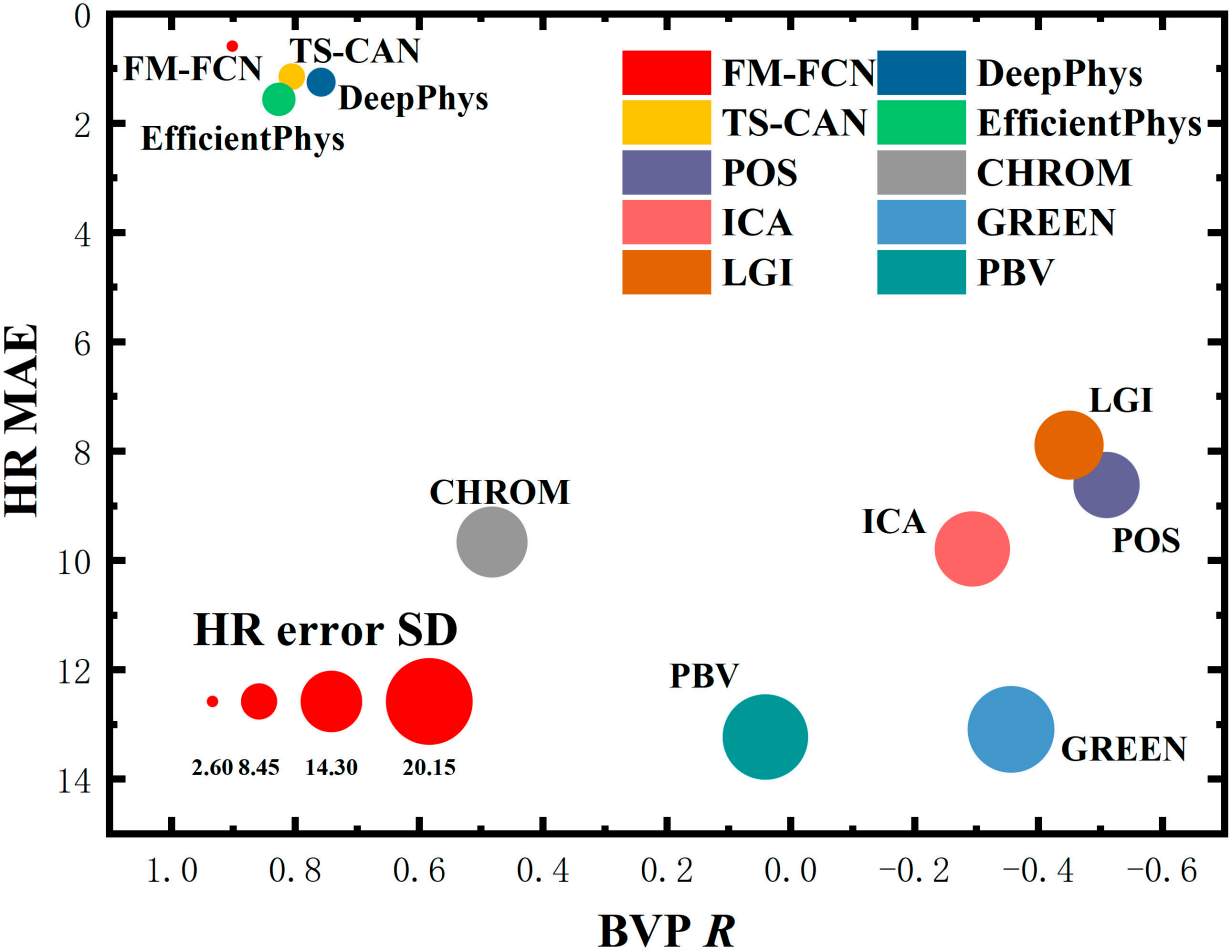
Comprehensive evaluation of various methods using bubble charts. The optimal performance region is located in the top left corner, with the bubble diameter indicative of the HR *SD*.

Furthermore, Fig. 8 clearly illustrates the disparities in BVP extraction quality and HR accuracy among various methods. The diagram highlights that the optimal position in the upper left quadrant correlates with the lowest HR error and the highest agreement between rPPG-derived eBVP waveforms and the rBVP waveforms, where the circle’s size reflects the HR error’s *SD*. This quadrant is indicative of the algorithms with the most comprehensive performance, underscoring the superiority of deep learning techniques over traditional methods in both BVP quality and HR accuracy. Among these deep learning algorithms, FM-FCN stands out for its exceptional performance, securing the top left position in the bubble chart. Detailed findings are presented in Table 2. Specifically, FM-FCN achieves the highest correlation with the reference signal in BVP waveform extraction accuracy and shows exceptional superiority in *SNR*, marking over a 50% improvement relative to other techniques. Regarding HR accuracy metrics, nearly 98.64% of HR estimates from FM-FCN align with the clinical accuracy criteria as stipulated in [28]. In addition, the mean errors (*MAE *and *MRE*) for HR calculated by FM-FCN are substantially lower—less than half—than those recorded by competing methods, signifying markedly superior accuracy. FM-FCN also exhibits remarkable robustness, as evidenced by its substantially reduced *SD *in comparison to other algorithms, ranging between 33.56% and 42.44% of the values noted for other methods.

**Table 2.** Quantitative statistics for training on UBFC-rPPG and testing on PURE

Method	BVP			HR					
	*MAE*	*SNR*	*R*	*MAE*	*MRE*	*SD*	*R*	* ACC*	* LoA* ([*L*, *U*])
**FM-FCN** (**ours**)	**0.30**	**9.21**	**0.90**	**0.59**	**1.05%**	**2.61**	**0.99**	**98.64%**	**[−4.92, 5.51]**
DeepPhys [35]	0.54	3.97	0.76	1.25	1.59%	6.72	0.96	97.50%	[−13.87, 12.83]
TS-CAN [37]	0.46	5.28	0.81	1.15	1.68%	6.15	0.96	98.18%	[−12.41, 12.11]
EfficientPhys [38]	0.42	6.35	0.83	1.56	2.06%	7.78	0.94	95.23%	[−15.94, 15.12]
POS [12]	1.42	−4.74	−0.51	8.6173	15.82%	15.38	0.73	75.45%	[−25.46, 38.65]
CHROM [27]	0.79	0.26	0.48	9.66	17.40%	16.51	0.67	71.36%	[−28.72, 41.78]
ICA [13]	1.30	−3.85	−0.29	9.7878	12.85%	17.51	0.55	66.36%	[−42.13, 35.45]
GREEN [11]	1.35	−4.23	−0.36	13.0877	16.42%	20.14	0.30	53.18%	[−51.72, 39.16]
LGI [15]	1.41	−4.55	−0.45	7.8902	10.52%	15.99	0.66	71.59%	[−37.16, 32.01]
PBV [31]	1.09	−2.29	0.04	13.2275	18.53%	19.83	0.41	56.82%	[−48.15, 45.15]

Notes: Considering the variation in PPG intensity across different parts, the normalization and band-pass filtering of both the rBVP waveforms and the eBVP waveforms are carried out before HR computation. *L* = mean – 1.96 × *SD* and *U* = mean + 1.96 × *SD*.

### Evaluation on large datasets

In this subsection, RLAP dataset is divided into a training set, which accounted for 75% of the data, and a test set for intradataset testing, consisting of the remaining 25%. The trained models are also cross-validated on PURE, UBFC-rPPG, and COHFACE datasets.

Table 3 provides a comprehensive overview of the findings from the intradataset testing. The results highlight the superiority of deep-learning-based approaches over classical-theory-based methods in terms of both BVP waveform quality and HR accuracy metrics. Notably, among the deep-learning-based approaches, FM-FCN stands out as the top performer. In relation to the quality of the BVP waveform, FM-FCN achieves the top rank in *MAE*, *SNR*, and *R*. It notably exhibits a significant 21.5% reduction in *MAE* compared to the second-ranked 3D-CAN model. Regarding HR estimation, FM-FCN also secures the top rank in *MAE*, *MRE*, *SD*, *R*, *ACC*, and *LoA*. In comparison to the second-ranked EfficientPhys model, FM-FCN showcases a remarkable 32.6% reduction in *MAE* for HR and a 25% reduction in the width of the *LoA* interval. Overall, FM-FCN performs exceptionally well, delivering outstanding outcomes in terms of accuracy and robustness across the evaluated metrics.

**Table 3.** Intradataset testing on RLAP

Method	BVP			HR					
	*MAE*	*SNR*	*R*	*MAE*	*MRE*	*SD*	*R*	*ACC*	*LoA*
**FM-FCN** (**ours**)	**0.36**	**7.93**	**0.87**	**0.97**	**1.31%**	**3.16**	**0.94**	**96.54%**	**[−6.71, 6.21]**
DeepPhys [35]	0.70	1.67	0.62	2.87	3.73%	6.82	0.75	88.27%	[−15.97, 11.94]
3D-CAN [37]	0.45	5.51	0.82	1.93	2.88%	4.71	0.84	92.14%	[−9.85, 10.11]
TS-CAN [37]	0.56	3.59	0.73	1.78	2.33%	4.80	0.87	93.16%	[−10.87, 8.78]
EfficientPhys [38]	0.51	4.40	0.77	1.44	1.91%	4.22	0.90	94.30%	[−9.35, 7.89]
POS [12]	1.44	−4.69	−0.49	3.15	4.28%	6.52	0.75	88.09%	[−13.65, 14.65]
CHROM [27]	0.83	−0.08	0.45	4.16	5.73%	8.08	0.64	83.49%	[−17.23, 18.33]
ICA [13]	1.23	−3.23	−0.16	7.79	10.17%	10.24	0.42	64.50%	[−28.24, 18.20]
GREEN [11]	1.29	−3.90	−0.25	11.36	14.99%	11.05	0.29	46.77%	[−34.86, 21.07]
LGI [15]	1.42	−4.55	−0.45	6.11	7.92%	9.64	0.54	73.12%	[−25.10, 15.71]
PBV [31]	1.18	−2.88	−0.08	8.42	11.09%	10.80	0.39	62.38%	[−29.99, 20.01]

To assess the robustness of various techniques, we conduct tests on models trained with RLAP dataset across 3 distinct datasets: UBFC-rPPG, COHFACE, and PURE.

Table 4 presents the experimental results obtained using UBFC-rPPG. Most of the approaches exhibit satisfactory performance in terms of both BVP waveform reconstruction capability and HR accuracy. This notable achievement can be attributed to the favorable conditions facilitated by UBFC-rPPG, where individuals are instructed to maintain a fixed distance from the camera with minimal movement, resulting in high-quality raw videos. Nevertheless, FM-FCN outperforms all other approaches and achieves the highest performance ranking.

**Table 4.** Cross-datasets testing on UBFC-rPPG

Method	BVP			HR					
	*MAE*	*SNR*	*R*	*MAE*	*MRE*	*SD*	*R*	*ACC*	*LoA*
**FM-FCN** (**ours**)	**0.35**	**7.76**	**0.88**	**0.48**	**0.51%**	**1.87**	**0.99**	**98.96%**	**[−3.91, 3.63]**
DeepPhys [35]	0.51	4.66	0.77	1.74	1.79%	7.16	0.92	95.83%	[−15.52, 12.94]
3D-CAN [37]	0.77	1.03	0.49	22.36	19.96%	20.05	−0.07	39.58%	[−61.96, 17.75]
TS-CAN [37]	0.52	4.23	0.76	1.43	1.47%	5.23	0.95	95.49%	[−11.33, 9.65]
EfficientPhys [38]	0.39	6.90	0.85	0.57	0.60%	2.01	0.99	98.96%	[−4.21, 3.96]
POS [12]	1.53	−5.03	−0.61	2.70	2.73%	7.81	0.89	94.10%	[−17.28, 14.72]
CHROM [27]	0.68	1.68	0.61	3.34	3.28%	9.22	0.86	92.36%	[−20.86, 16.71]
ICA [13]	1.37	−3.90	−0.36	11.74	11.26%	20.41	0.51	73.26%	[−51.81, 29.78]
GREEN [11]	1.43	−4.57	−0.45	15.89	15.14%	22.41	0.40	62.85%	[−60.11, 29.98]
LGI [15]	1.47	−4.76	−0.52	13.06	12.39%	21.23	0.46	69.79%	[−54.81, 30.68]
PBV [31]	1.24	−3.10	−0.17	15.04	14.13%	22.33	0.39	66.67%	[−59.05, 30.78]

The results of the experiments conducted using the COHFACE dataset are presented in Table 5. It is worth noting that the COHFACE dataset has a high compression rate [29], leading to a lower *SNR*. FM-FCN achieves the highest accuracy in HR estimation with an *ACC* value of 73.19% and an *MAE* of 5.91, as well as ranking first in terms of the quality of reconstructed BVP waveforms. These findings indicate that FM-FCN demonstrates better robustness even when dealing with low-quality video data.

**Table 5.** Cross-datasets testing on COHFACE

Method	BVP			HR					
	*MAE*	*SNR*	*R*	*MAE*	*MRE*	*SD*	*R*	*ACC*	*LoA*
**FM-FCN** (**ours**)	**0.75**	**0.75**	**0.52**	**5.91**	**7.64%**	9.73	0.58	**73.19%**	[−24.94, 16.62]
DeepPhys [35]	0.90	−0.86	0.35	5.97	7.71%	**9.57**	**0.61**	72.88%	**[−24.78, 15.60]**
3D-CAN [37]	1.02	−1.91	0.18	31.49	28.14%	20.75	−0.18	16.49%	[−73.26, 11.42]
TS-CAN [37]	0.89	−0.88	0.35	8.43	11.02%	11.52	0.36	63.79%	[−31.31, 20.50]
EfficientPhys [38]	0.86	−0.49	0.39	8.04	10.69%	10.93	0.43	64.41%	[−29.58, 20.66]
POS [12]	1.15	−3.20	−0.06	19.82	22.12%	17.79	0.09	39.29%	[−51.89, 52.54]
CHROM [27]	1.04	−2.51	0.08	19.15	21.99%	18.00	0.04	42.37%	[−49.41, 53.38]
ICA [13]	1.15	−3.09	−0.05	21.99	22.58%	18.91	0.07	34.67%	[−63.77, 39.74]
GREEN [11]	1.23	−3.65	−0.18	36.40	34.29%	24.28	−0.10	18.80%	[−89.17, 22.93]
LGI [15]	1.15	−3.25	−0.07	38.56	35.92%	22.93	−0.05	12.33%	[−87.78, 14.94]
PBV [31]	1.08	−2.68	0.05	40.59	38.51%	20.96	−0.06	5.55%	[−88.76, 13.87]

**Fig. 9.**
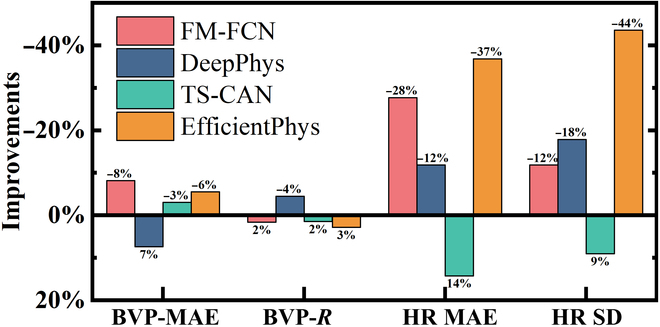
Comparative performance improvements of models trained on the RLAP dataset versus those trained on the UBFC-rPPG dataset. The improvement metrics are calculated by subtracting the values listed in Table 6 from their counterparts in Table 2, and then dividing by the values in Table 2.

Table 6 showcases the experimental results on the PURE dataset, using deep learning models trained on the RLAP dataset, contrasting with the UBFC-rPPG dataset used in Table 2. The table clearly demonstrates that FM-FCN retains superior performance across all evaluated metrics, highlighting its stability and robustness regardless of the training dataset used. To further investigate the performance differences between the 2 datasets, we calculate the relative improvement, with findings illustrated in Fig. 9. This analysis shows enhanced BVP waveform quality and HR accuracy in models trained on RLAP compared to those trained on UBFC-rPPG. Using *t* tests, we quantitatively assess the variance in model performance between the datasets, as detailed in Table 7. Except for the DeepPhys model, all models show significant improvements in BVP waveform extraction quality. However, improvements in HR accuracy are not statistically significant, likely because of the simplicity of the HR calculation model. The RLAP-trained models’ superiority over the UBFC-rPPG-trained models is attributed to a richer collection of motion scene data, which bolsters model robustness. This emphasizes the critical role of dataset comprehensiveness in improving model performance. In summary, FM-FCN exhibits notable accuracy and robustness when trained on datasets of varying scales (RLAP and UBFC-rPPG), showcasing its strong generalization capabilities and exceptional performance across diverse datasets.

**Table 6.** Cross-datasets testing on PURE

Method	BVP			HR					
	*MAE*	*SNR*	*R*	*MAE*	*MRE*	*SD*	*R*	*ACC*	*LoA*
**FM-FCN** (**ours**)	**0.28**	**10.06**	**0.92**	**0.4275**	**0.65%**	**2.30**	**0.99**	**99.32%**	**[−4.64, 4.54]**
DeepPhys [35]	0.58	3.29	0.72	1.1026	1.48%	5.52	0.97	98.18%	[−11.29, 10.73]
3D-CAN [37]	0.66	2.61	0.60	13.5871	19.61%	19.09	0.12	58.86%	[−45.03, 46.78]
TS-CAN [37]	0.45	5.56	0.82	1.3104	1.78%	6.71	0.96	97.05%	[−13.69, 13.08]
EfficientPhys [38]	0.39	6.90	0.85	0.9868	1.66%	4.39	0.98	97.50%	[−8.68, 8.95]

**Fig. 10.**
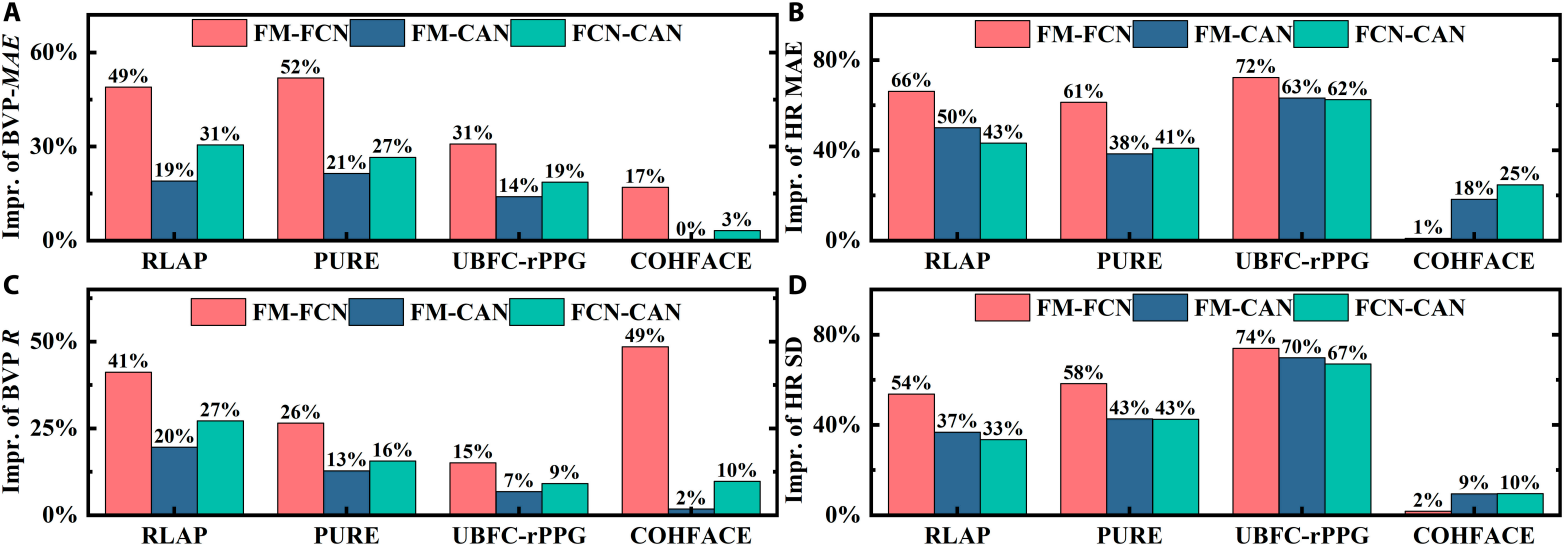
Performance improvements across various modules of the FM-FCN, demonstrated through enhancements in BVP *MAE*, HR *MAE*, BVP *R*, and HR *SD*. (A) The improvement of BVP *MAE*. (B) The improvement of HR *MAE*. (C) The improvement of BVP *R*. (D) The improvement of HR *SD*. Impr. is short for improvement.

**Table 7.** Significance analysis of model performance: RLAP versus UBFC-rPPG trained models via *t* test (*α* = 0.05). A value of 1 indicates a significant difference, while 0 denotes no significant difference between models. The term “CI” represents the confidence interval, highlighting the magnitude and direction of significant changes. HR error denotes the error between eHR and rHR.

Method	BVP*R*			HR *Error*		
	*h*	*P*	CI	*h*	*p*	CI
**FM-FCN** (**ours**)	**1**	1.55 × 10^−7^	[0.009, 0.020]	0	0.28	[−0.46, 0.13]
DeepPhys [35]	**1**	2.09 × 10^−22^	[−0.04, −0.027]	0	0.49	[−0.57, 0.27]
TS-CAN [37]	**1**	2.69 × 10^−4^	[0.006, 0.019]	0	0.56	[−0.38, 0.71]
EfficientPhys [38]	**1**	3.89 × 10^−11^	[0.017, 0.030]	0	0.11	[−1.28, 0.13]

To quantitatively assess the performance enhancements of FM-FCN in rPPG tasks relative to other models, we utilize *t* tests to analyze significant differences in key performance metrics. Table 8 demonstrates that FM-FCN achieves substantial improvements in the *MAE* of BVP waveforms and HR accuracy, indicating marked reductions in both *MAE* and HR errors when compared with competing models. Furthermore, FM-FCN significantly surpasses other models in the correlation (*R*) of BVP waveforms and in *SNR*, with confidence intervals revealing significant enhancements in both *SNR* and *R*. The outcomes of this significance analysis for the PURE, COHFACE and RLAP datasets are detailed in Tables S1 to S3, respectively. These *t* test findings further validate the superiority of FM-FCN in overcoming rPPG detection challenges.

**Table 8.** Pair-wise *t*-test comparative analysis of significant performance improvements of FM-FCN over other models on UBFC-rPPG (*α* = 0.05). The analysis involves *t* tests between FM-FCN and each of the other models.

FM-FCN vs.		DeepPhys [35]	3D-CAN [37]	TS-CAN [37]	EfficientPhys [38]
**BVP *MAE***	***P*(*h*)**	1(1.17 × 10^−43^)	1(2.84 × 10^−78^)	1(1.15 × 10^−54^)	1(2.49 × 10^−9^)
	**CI**	[−0.18, −0.14]	[−0.44, −0.38]	[−0.19, −0.15]	[−0.05, −0.03]
**BVP *SNR***	***P*(*h*)**	1(9.89 × 10^−46^)	1(1.36 × 10^−82^)	1(1.37 × 10^−55^)	1(2.63 × 10^−9^)
	**CI**	[2.75, 3.46]	[6.26, 7.22]	[3.19, 3.89]	[0.59, 1.14]
**BVP *R***	***P*(*h*)**	1(2.45 × 10^−36^)	1(1.26 × 10^−70^)	1(2.89 × 10^−49^)	1(6.92 × 10^−10^)
	**CI**	[0.10, 0.13]	[0.36, 0.43]	[0.11, 0.13]	[0.02, 0.04]
**HR error**	***P*(*h*)**	1(1.97 × 10^−3^)	1(3.64 × 10^−50^)	1(1.26 × 10^−3^)	1(7.93 × 10^−3^)
	**CI**	[−2.05, −0.47]	[−24.23, −19.52]	[−1.52, −0.37]	[−0.15, −0.02]

### Ablation study

FM-FCN is an advanced extension of DeepPhys (CAN). To evaluate the impact of distinct modules integrated into FM-FCN, we have undertaken an ablation study. In this subsection, we introduce 2 variations: FM-CAN, which incorporates FM into DeepPhys, and FCN-CAN, which replaces DeepPhys’ fully connected layer with FCN. These variations are rigorously trained on 75% of the RLAP dataset and subsequently assessed on the remaining 25%, along with additional datasets such as PURE, UBFC-rPPG, and COHFACE. This methodology allows for a thorough examination of the enhancements and contributions each module brings to the FM-FCN’s performance.

Table 9 offers a detailed analysis from an ablation study, comparing the performance enhancements of FM-FCN, FM-CAN, and FCN-CAN against DeepPhys. In terms of BVP quality metrics, implementing FM and FCN separately yields reductions in *MAE* of 21% and 27%, respectively, and substantial improvements in the *SNR* by 62% and 83%. The combined FM-FCN framework notably reduces *MAE* by 52% and increases *SNR* by an impressive 206%. Regarding HR accuracy metrics, both FM and FCN contribute to *MAE* reductions of 38% and 41%, respectively, and achieve a 42% narrowing in the *LoA *width. The integration of FM and FCN enhances these effects, leading to an outstanding 61% reduction in *MAE* and a significant 58% decrease in *LoA *width. These results strongly support the essential contributions of FM and FCN to improving the accuracy and reliability of rPPG measurements.

**Table 9.** Ablation study of FM-FCN on PURE.

Method	BVP			HR		
	*MAE*	*SNR*	*R*	*MAE*	*SD*	*LoA*
**FM-FCN**	**0.28**	**10.06**	**0.92**	**0.43**	**2.30**	**[−4.64, 4.54]**
FM-CAN	0.46	5.32	0.82	0.68	3.16	[−6.46, 6.22]
FCN-CAN	0.43	6.01	0.84	0.65	3.17	[−6.45, 6.23]
DeepPhys (CAN)	0.58	3.29	0.72	1.10	5.52	[−11.29, 10.73]

The results for the RLAP, UBFC-rPPG, and COHFACE datasets are provided in Tables S4 to S6, respectively. Figure 10 shows the improvement statistics for BVP *MAE*, HR *MAE*, BVP *R*, and HR *SD*. It is evident that both FM and FCN modules significantly enhance the waveform quality of BVP, with FM-FCN showing a more pronounced improvement. However, FM-FCN performs relatively worse than FM-CAN and FCN-CAN on COHFACE. A significant factor contributing to this discrepancy is the video compression by COHFACE, which leads to considerable rPPG signal loss. In scenarios with degraded signal quality, there is a need for parallel optimization of HR computation algorithms. Nonetheless, a consistent approach utilizing the fast Fourier transform to analyze the pulse signal spectrum for HR calculation has been implemented across all experiments in this work. Overall, the FM and FCN modules greatly enhance both the BVP waveform extraction and the accuracy of HR computation across the network. The marked improvement following the FM-FCN combination preliminarily suggests its efficacy for rPPG applications.

**Fig. 11.**
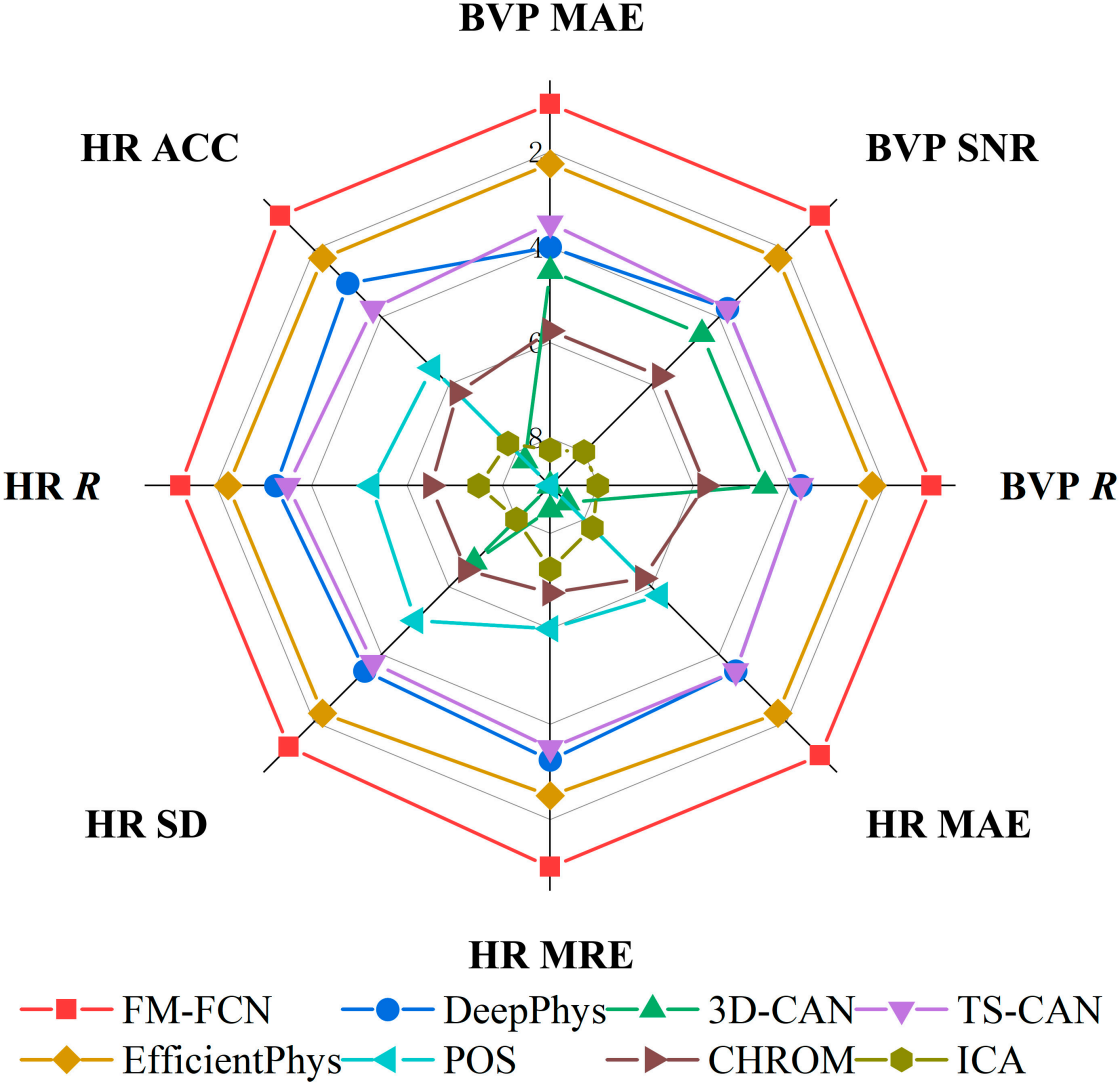
Overall performance ranking of different rPPG approaches.

## Discussion

In this work, we propose FM-FCN for processing physiological signals. The FCN component serves as the foundation for multiframe signal processing in the temporal dimension, aligning with the periodic characteristics of signal processing. By fusing classical signal processing techniques with deep learning technology, FM effectively suppresses noise and enhances the signal. The significance of these 2 modules is demonstrated through ablation experiments.

The results obtained from testing on both small-scale and large-scale datasets provide comprehensive evidence of the generalization ability and robustness of FM-FCN. To further support our conclusions, we conduct rankings based on the results presented in Tables 2 to 6, and the average rankings consistently place FM-FCN in the top position. For a more intuitive performance comparison, Fig. 11 visually illustrates that FM-FCN achieves close to or the best results in each metric. Notably, FM-FCN shows considerable improvements in both BVP and HR compared to the second-ranked method. Specifically, the quality of BVP waveform reconstruction sees a decrease of 20.23% in *MAE *and an increase of 79.95% in *SNR*. In terms of HR accuracy index, there is a decrease of 35.85% in *MAE*, 29.65% in *SD*, and 32.88% in *LoA* width. These achievements can be attributed not only to FM but also to the design of FCN. By replacing fully connected layers with FCN, we reduce the number of parameters and enable effective weight parameter sharing, leveraging temporal information correlations.

In summary, FM-FCN is an innovative approach to processing physiological signals. Its smaller scale of model parameters, enhanced accuracy, and robustness renders it a promising method in the realm of rPPG and a potential tool in various physiological fields.

## Conclusion and Future Works

The amplitude and morphology exhibiting periodicity are typical characteristics of physiological signals. The presence of noise is a key challenge that interferes with the extraction of physiological signals. In this work, we propose a novel FM-FCN that fuses classical signal processing theory with deep learning techniques, enabling FM-FCN to retain spatial extraction capabilities while incorporating the fusion ability of temporal information. The fusion of temporal information enhances the model’s resistance to noise interference. Specifically, by replacing fully connected layers with FCN, FM-FCN achieves parameter reduction and enhanced parameter sharing. Moreover, FCNs are better suited to capture the periodic characteristics of signals. By utilizing input data with a duration of at least twice the signal’s period and combining it with FM, FM-FCN effectively leverages temporal correlation and achieves higher-quality extraction of physiological signals. Experimental evaluations affirm that FM-FCN significantly improves BVP waveform extraction quality and HR accuracy across various datasets. Overall, FM-FCN, which comprehensively considers the periodicity and temporal correlation of physiological signals by effectively fusing traditional signal processing technology with deep learning, holds promise as an important tool for processing physiological periodic data.

In our future work, we will design a multitask network to simultaneously collect HR, breathing, and other variables. In addition, we plan to combine FM with other convolutional networks to maximize the utilization of temporal dimension information while minimizing parameter costs.

## Methods

### Overview of models

Classical-theory-based rPPG

The main steps of classical-theory-based rPPG are as follows: (a) face detection; (b) extraction of the region of interest (ROI), typically areas rich in capillaries; (c) signal preprocessing and color transform; (d) signal postprocessing and BVP signal reconstruction; and, (e) eventually, estimation of vital signs signal. Verkruysse et al. [3] conducted a study on estimating HR using facial videos and validating its feasibility. However, the ROI was manually selected using bounding boxes. Poh et al. [11] introduced an automatic BVP extraction technique that utilizes automatic face tracking and BSS to decompose color channels into independent components, enabling automatic extraction of BVP signals from facial videos. Lewandowska et al. [30] achieved similar accuracy as ICA-based methods with lower computational complexity by extracting “pulse” components using principal components analysis. Haan and Jeanne [27] developed chrominance-based rPPG methods that exploit color changes caused by blood flow. These methods outperformed BSS-based approaches in terms of accuracy. De Haan and Van Leest [31] proposed a technique to map the PPG image signals by linear combination of red–green–blue data. This mapping is designed to be orthogonal to motion-induced artifacts, resulting in superior motion robustness. Pilz et al. [15] leverage LGI to enhance HR estimation from facial videos in uncontrolled environments by introducing features invariant to local transformations, thus concentrating the blood volume signal energy distribution. These advancements have significantly contributed to the theoretical foundation and evolution of rPPG technology.

While classical-theory-based rPPG techniques have shown promising results under normal ambient light conditions, they may face challenges and limitations under complex conditions, such as noise interference and model limitations. These limitations arise from the loss of critical information [6], leading to less accurate estimation of vital signs. Deep-learning-based methods provide an alternative approach to addressing these limitations and improving the quality of BVP waveforms for better estimation of vital signs.

### Deep-learning-based rPPG

In recent years, there has been a growing interest in deep-learning-based rPPG technologies. This can be attributed to the powerful capabilities of deep learning techniques in spatial decomposition, adaptive information extraction, and information reconstruction [32,33].

By utilizing well-designed network models, deep learning algorithms have shown effectiveness in restoring high-quality BVP signals. Some notable examples include HR-CNN [34], which uses a 2-step CNN to extract the rPPG signal and then feeds it to a one-dimensional (1D) CNN for HR estimation. DeepPhys captures physiological motions in videos by calculating normalized frame differences based on the skin reflection model and improves motion estimation using attention from human appearance in neural networks [35]. PhysNet introduces a 3D network that accurately recovers BVP signals from facial videos by considering temporal context [36]. TS-CAN leverages temporal shift modules (TSMs) to perform efficient temporal modeling and remove various sources of noise without additional computational overhead [37]. EfficientPhys creates a preprocessing-free neural architecture that is simple to use and deploy, efficient on mobile devices, and accurate on settings with various types of noise [38].

BVP signal, being a temporal signal, serves as the foundation for extracting other vital sign signals. While CNN networks such as HR-CNN and DeepPhys excel at capturing spatial information and demonstrating robustness in different motion scenarios, they may be weak in incorporating temporal information. However, the integration of temporal information is the crucial for accurate waveform reconstruction. PhysNet addresses this by incorporating temporal information through a 3D-CNN model, but this increases complexity and affects real-time performance. TS-CAN, on the other hand, reduces complexity while utilizing temporal information through TSM. However, its effectiveness lies in video action recognition using a random movement strategy, with limited improvement on extracting periodic physiological signals. EfficientPhys comprises both convolution-based and transformer-based models, achieving significant improvements in HR accuracy. The performance of the transformer-based variant of EfficientPhys is not as robust as that of its convolution-based counterpart, largely due to a scarcity of pretraining data. Consequently, references to EfficientPhys in subsequent experiments are specifically directed toward the convolution-based variant. However, it is important to note that all EfficientPhys variants incorporate temporal information across a limited frames through the TSM, which results in a constrained and arbitrary integration of temporal data. In this work, we aim to develop a novel spatiotemporal network model for periodic physiological signals.

### Spatiotemporal networks

Spatial and temporal information constitute 2 fundamental data types essential for understanding the real world [39,40]. Spatial data delineate the physical locations or positions of objects, facilitating the analysis of patterns, spatial relationships, proximity, accessibility, and distributions. Conversely, temporal data offer insights into the timing of events and how phenomena change over time, pivotal for grasping processes, trends, and evolving patterns.

Deep learning has provided significant advantages in image spatial data processing tasks, such as texture filtering [41,42], image dehazing [43], and edge-preserving smoothing [44], among others [45]. This success has sparked increased interest in merging spatial and temporal data to bolster performance in video or sequential data tasks. Leading methodologies include 3D-CNNs [46], 2-stream network frameworks [16], LSTM networks [7], attention mechanisms [35], temporal relation network [47], and TSM [48]. Each method not only offers distinct advantages but also comes with inherent limitations. For instance, while 3D-CNNs are adept at learning spatiotemporal features, they may be prone to overfitting or fail to converge. Two-stream networks and attention mechanisms offer layered integration of spatial and temporal data, albeit confined to specific network locations. LSTMs are effective in processing temporal data but are complex to train and may falter with long-term dependencies. Meanwhile, temporal relation network and TSM maintain lower complexity and adeptly map relationships within temporal sequences yet might not fully leverage signals’ inherent properties, like periodicity.

Physiological signals are characterized by gradual changes, periodic patterns, and repetitive morphology, presenting unique opportunities for signal extraction from interference-rich data. Classical signal processing techniques, which harness these time-domain characteristics, have been instrumental in isolating weak signals. This work aims to design an innovative spatiotemporal network that marries classical signal processing with deep learning technologies, tailored for the efficient extraction of physiological signals.

## Data Availability

The codes and datasets are available online at https://github.com/zhaoqi106/FM-FCN.
