# Supplementary material for: FM-FCN: A Neural Network with Filtering Modules for Accurate Vital Signs Extraction
Source: Research (Wash D C). 2024 May 10;7:0361. doi: 10.34133/research.0361 (PMC11082448; doi:10.34133/research.0361)
Supplement: Supplementary 1 — Tables S1 to S6 [file research.0361.f1.docx]

**Supplementary Materials**

**Table. S1**. Pair-wise t-test comparative analysis of significant performance improvements of FM-FCN over other models on PURE ($\alpha=0.05$).

| **FM-FCN v.s.** | | DeepPhys [28] | 3D-CAN [30] | TS-CAN [30] | EfficientPhys [31] |
| --- | --- | --- | --- | --- | --- |
| **BVP *MAE*** | ***p*(*h*)** | 1(3.77e-187) | 1(1.61e-130) | 1(1.37e-130) | 1(1.31e-83) |
|  | ***ci*** | [-0.31,-0.29] | [-0.40,-0.36] | [-0.18,-0.16] | [-0.12,-0.11] |
| **BVP *SNR*** | ***p*(*h*)** | 1(3.16e-161) | 1(4.55e-157) | 1(1.48e-122) | 1(4.32e-92) |
|  | ***ci*** | [6.47,7.08] | [7.10,7.79] | [4.24,4.77] | [2.92,3.40] |
| **BVP *R*** | ***p*(*h*)** | 1(7.93e-129) | 1(7.92e-88) | 1(1.15e-90) | 1(1.60e-41) |
|  | ***ci*** | [0.18,0.20] | [0.29,0.34] | [0.09,0.11] | [0.06,0.07] |
| **HR Error** | ***p*(*h*)** | 1(5.41e-03) | 1(1.24e-38) | 1(3.76e-03) | 1(2.79e-03) |
|  | ***ci*** | [-1.15,-0.20] | [-14.96,-11.36] | [-1.48,-0.29] | [-0.92,-0.19] |

**Table. S2.** Pair-wise t-test comparative analysis of significant performance improvements of FM-FCN over other models on COHFACE ($\alpha=0.05$).

| **FM-FCN v.s.** | | DeepPhys [28] | 3D-CAN [30] | TS-CAN [30] | EfficientPhys [31] |
| --- | --- | --- | --- | --- | --- |
| **BVP *MAE*** | ***p*(*h*)** | 1(3.53e-109) | 1(5.07e-175) | 1(1.57e-117) | 1(1.94e-88) |
|  | ***ci*** | [-0.16,-0.14] | [-0.29,-0.26] | [-0.16,-0.14] | [-0.12,-0.10] |
| **BVP *SNR*** | ***p*(*h*)** | 1(1.67e-93) | 1(1.60e-141) | 1(4.17e-109) | 1(3.19e-82) |
|  | ***ci*** | [1.48,1.73] | [2.50,2.82] | [1.50,1.74] | [1.13,1.34] |
| **BVP *R*** | ***p*(*h*)** | 1(1.93e-93) | 1(2.28e-165) | 1(4.33e-107) | 1(3.71e-80) |
|  | ***ci*** | [0.16,0.18] | [0.32,0.36] | [0.16,0.19] | [0.12,0.14] |
| **HR Error** | ***p*(*h*)** | 0(8.80e-01) | 1(6.51e-147) | 1(6.02e-10) | 1(8.58e-08) |
|  | ***ci*** | [-0.81,0.69] | [-27.05,-24.11] | [-3.31,-1.73] | [-2.90,-1.36] |

**Table. S3.** Pair-wise t-test comparative analysis of significant performance improvements of FM-FCN over other models on RLAP ($\alpha=0.05$).

| **FM-FCN v.s.** | | DeepPhys [28] | 3D-CAN [30] | TS-CAN [30] | EfficientPhys [31] |
| --- | --- | --- | --- | --- | --- |
| **BVP *MAE*** | ***p*(*h*)** | 1(0.00e+00) | 1(4.79e-189) | 1(0.00e+00) | 1(0.00e+00) |
|  | ***ci*** | [-0.345,-0.335] | [-0.10,-0.09] | [-0.21,-0.20] | [-0.16,-0.15] |
| **BVP *SNR*** | ***p*(*h*)** | 1(0.00e+00) | 1(7.72e-235) | 1(0.00e+00) | 1(0.00e+00) |
|  | ***ci*** | [6.15,6.37] | [2.30,2.57] | [4.25,4.44] | [3.44,3.63] |
| **BVP *R*** | ***p*(*h*)** | 1(0.00e+00) | 1(7.67e-86) | 1(0.00e+00) | 1(0.00e+00) |
|  | ***ci*** | [0.25,0.26] | [0.05,0.06] | [0.13,0.14] | [0.09,0.10] |
| **HR Error** | ***p*(*h*)** | 1(2.40e-64) | 1(4.68e-25) | 1(8.57e-27) | 1(1.21e-12) |
|  | ***ci*** | [-2.12,-1.69] | [-1.14,-0.78] | [-0.95,-0.66] | [-0.60,-0.34] |

**Table. S4.** Ablation study of FM-FCN on RLAP.

| **Method** |  | **BVP** | | |  | **HR** | | |
| --- | --- | --- | --- | --- | --- | --- | --- | --- |
|  |  | ***MAE*** | ***SNR*** | ***R*** |  | ***MAE*** | ***STD*** | ***LoA*** |
| **FM-FCN** |  | **0.36** | **7.93** | **0.87** |  | **0.97** | **3.16** | **[-6.71,6.21]** |
| FM-CAN |  | 0.56 | 3.55 | 0.74 |  | 1.44 | 4.31 | [-9.59,7.93] |
| FCN-CAN |  | 0.48 | 5.07 | 0.78 |  | 1.63 | 4.54 | [-10.18,8.39] |
| DeepPhys(CAN) |  | 0.70 | 1.67 | 0.62 |  | 2.87 | 6.82 | [-15.97,11.94] |

**Table. S5.** Ablation study of FM-FCN on UBFC-rPPG.

| **Method** |  | **BVP** | | |  | **HR** | | |
| --- | --- | --- | --- | --- | --- | --- | --- | --- |
|  |  | ***MAE*** | ***SNR*** | ***R*** |  | ***MAE*** | ***STD*** | ***LoA*** |
| **FM-FCN** |  | **0.35** | **7.76** | **0.88** |  | **0.48** | **1.87** | **[-3.91,3.63]** |
| FM-CAN |  | 0.44 | 6.00 | 0.82 |  | 0.64 | 2.17 | [-4.63,4.20] |
| FCN-CAN |  | 0.41 | 6.48 | 0.84 |  | 0.65 | 2.36 | [-5.04,4.52] |
| DeepPhys(CAN) |  | 0.51 | 4.66 | 0.77 |  | 1.74 | 7.16 | [-15.52,12.94] |

**Table. S6.** Ablation study of FM-FCN on COHFACE.

| **Method** |  | **BVP** | | |  | **HR** | | |
| --- | --- | --- | --- | --- | --- | --- | --- | --- |
|  |  | ***MAE*** | ***SNR*** | ***R*** |  | ***MAE*** | ***STD*** | ***LoA*** |
| **FM-FCN** |  | **0.75** | **0.75** | **0.52** |  | **5.91** | **9.73** | **[-24.94,16.62]** |
| FM-CAN |  | 0.90 | -0.84 | 0.35 |  | 7.06 | 10.48 | [-27.78,17.22] |
| FCN-CAN |  | 0.87 | -0.59 | 0.39 |  | 7.44 | 10.48 | [-28.24,17.90] |
| DeepPhys(CAN) |  | 0.90 | -0.86 | 0.35 |  | 5.97 | 9.57 | [-24.78,15.60] |
